# Examining current or future trade-offs for biodiversity conservation in north-eastern Australia

**DOI:** 10.1371/journal.pone.0172230

**Published:** 2017-02-21

**Authors:** April E. Reside, Jeremy VanDerWal, Atte Moilanen, Erin M. Graham

**Affiliations:** 1 Centre for Tropical Environmental and Sustainability Sciences, College of Science and Engineering, James Cook University, Townsville, Queensland, Australia; 2 Centre for Tropical Biodiversity and Climate Change, College of Science and Engineering, James Cook University, Townsville, Queensland, Australia; 3 eResearch Centre, James Cook University, Townsville, Queensland, Australia; 4 Department of Biosciences, (Viikinkaari 1), University of Helsinki, Helsinki, Finland; Sichuan University, CHINA

## Abstract

With the high rate of ecosystem change already occurring and predicted to occur in the coming decades, long-term conservation has to account not only for current biodiversity but also for the biodiversity patterns anticipated for the future. The trade-offs between prioritising future biodiversity at the expense of current priorities must be understood to guide current conservation planning, but have been largely unexplored. To fill this gap, we compared the performance of four conservation planning solutions involving 662 vertebrate species in the Wet Tropics Natural Resource Management Cluster Region in north-eastern Australia. Input species data for the four planning solutions were: 1) current distributions; 2) projected distributions for 2055; 3) projected distributions for 2085; and 4) current, 2055 and 2085 projected distributions, and the connectivity between each of the three time periods for each species. The four planning solutions were remarkably similar (up to 85% overlap), suggesting that modelling for either current or future scenarios is sufficient for conversation planning for this region, with little obvious trade-off. Our analyses also revealed that overall, species with small ranges occurring across steep elevation gradients and at higher elevations were more likely to be better represented in all solutions. Given that species with these characteristics are of high conservation significance, our results provide confidence that conservation planning focused on either current, near- or distant-future biodiversity will account for these species.

## Introduction

Effective conservation planning and appropriate implementation are crucial for stemming the current biodiversity decline. However, the challenges increase when future, enigmatic threats such as climate change are considered. Given the uncertainty of the future, there is a lack of guidance on how to plan for future conservation priorities where these might come at the expense of current priorities. For example, investing in an area with high current biodiversity value may be a less-optimal solution compared to investing in another area with slightly lower biodiversity value currently, but likely to have the highest value in 50 years’ time. Examination of trade-offs between priorities for either current or future biodiversity, and ways of resolving those trade-offs are necessary to provide guidance for planners and managers.

With current greenhouse gas emissions, the globe is on track for severe climate change [1]. Moreover, species distributions are already shifting, many of them roughly in the expected direction to track their climatic niches [2, 3]. While current protected areas are important for the species they currently contain, and for species already moving in response to climate change [4–6], they are likely to be less effective for protecting species in the future, including for birds [7], amphibians [8] and moths [9]. These ongoing range shifts and reorganisation of assemblages result in a trade-off between investing in areas of high present conservation value versus areas of likely future conservation value [10]. For example, increasing the weight given to future conservation priority for amphibians and reptiles in Europe can result in substantial loss to current biodiversity priorities with only minor gains for future [11]. Studies show remarkable variation in consistency in the selection of priority biodiversity areas for current or projected future: from less than 2% of planning units remaining consistent for both current and future species of herptiles on the Iberian Peninsula [12], to 40% for corals surrounding Japan [13], to over 72% for mammalian carnivores globally [14]. It is therefore important to examine the potential biodiversity trade-offs through time to identify the species most likely to be represented by protected areas in the future and those that may require alternative conservation actions [6].

Methodologies that identify priority areas for conservation incorporating both current and future species distributions, and the connectivity between these time points have been developed [11, 15, 16]; and the impact of a range of uncertainties (e.g. from modelling parameters, unknown future climate or species response to future climates) have been examined [11, 17]. However, understanding which species are likely to benefit from conservation planning focussed on different time points is required. Furthermore, the characteristics of distributions that increase the likelihood of species benefitting from protected-area networks optimised for current versus future climates have yet to be quantified.

Climate change is a severe threat to biodiversity in north-eastern Australia, across rainforest, Eucalypt forest and savanna biomes [18–23]. For example, even a moderate increase in temperature is likely to severely impact the rainforest endemics of the Wet Tropics [18] and bird species on Cape York Peninsula [20, 23]. As a result, Natural Resource Management (NRM) groups in this region (http://www.nrm.gov.au/regional/regional-nrm-organisations) need to incorporate climate change into their spatial planning. Like managers elsewhere, the NRM groups want to plan for the best biodiversity outcomes if a severe climate-change future is realised, but without diverting scarce resources away from current conservation priorities unnecessarily [24]. Understanding which species benefit and which do not from the optimisation of conservation actions over multiple time points simultaneously (current, near- and distant-future) is required.

This study investigated whether there is a trade-off between planning for biodiversity conservation under current or future climates in north-eastern Australia. We examined the differences between conservation planning solutions to protect 662 terrestrial species, using current and two future distributions separately and combined. In addition, we investigated the characteristics of species distributions–such as extent, elevation and nestedness of future distributions within current ones–to find factors that determine how well species will be accounted for in any of the solutions. In particular, we determined for which species current distributions would be accounted for if only a future solution was used, and vice versa.

## Materials and methods

### Study area

**Fig 1:**
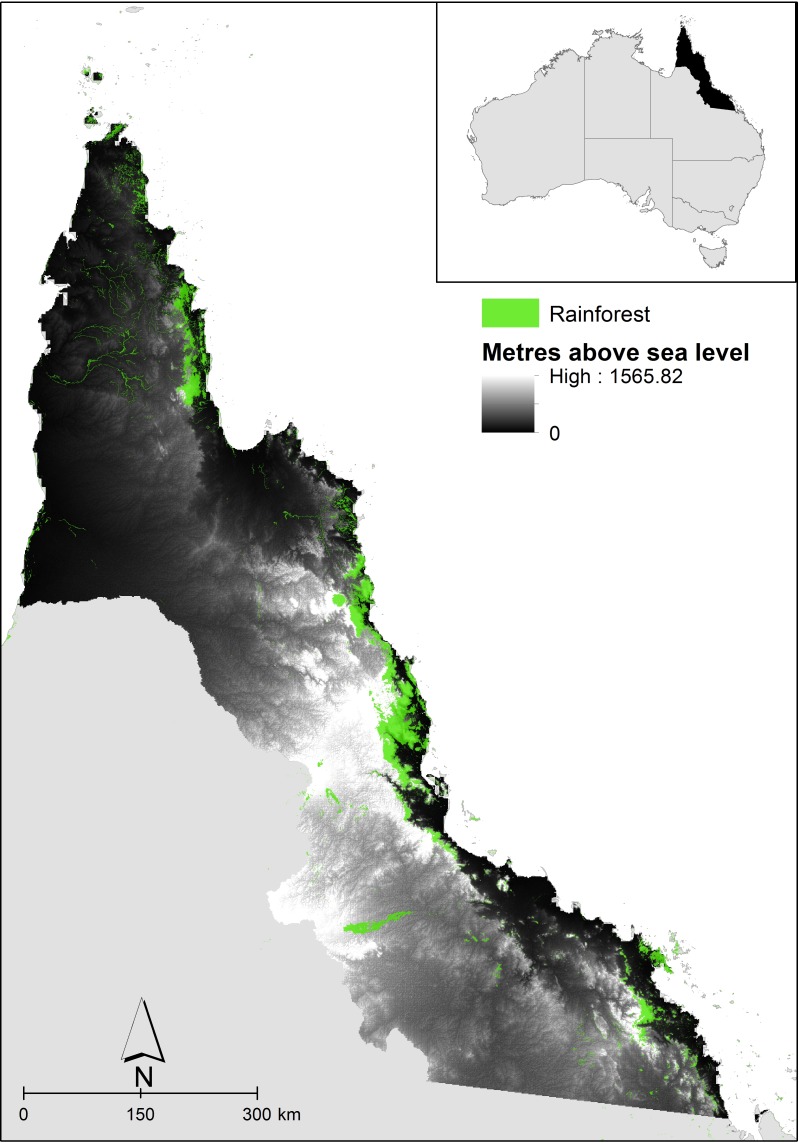
The Wet Tropics Natural Resource Management (NRM) Cluster region of north-eastern Australia. Elevation is shown in greyscale and rainforest extent in green.

The Wet Tropics NRM cluster region incorporates four NRM groups across the Torres Strait (Torres Strait Regional Authority), Cape York (Cape York NRM), the Wet Tropics (Terrain NRM) and Mackay-Whitsundays (Reef Catchments NRM) in north-eastern Australia (Fig 1). For this study, the Torres Strait region was not included as sufficient, reliable species data were not available. The mainland section of the region (i.e. the other three NRM jurisdictions) stretches from -10.12 to -23.53 degrees latitude, and consists of small rainforest patches clustered along the east coast, from sea level to 1565 m, bordered by wet Eucalypt forests out to open Eucalypt and other woodlands.

The rainforest regions have high numbers of endemic vertebrates, many with extremely restricted distributions [25–27]. The dry forests and woodlands are also of high biodiversity value, with some endemic vertebrates [28–32]. For this study, the extent of the NRM cluster region was buffered using the outlines of a broader catchment area, and all vertebrates with distributions overlapping the study extent were included [31]. There were species with varying conservation importance to the region, from species with small extents endemic to the region to those occurring widely across the continent and only marginally within the study area.

### Species distribution models

Locality data on vertebrate species were acquired from the Atlas of Living Australia (http://www.ala.org.au/), Queensland Museum (http://www.qm.qld.gov.au/), and the Centre for Tropical Biodiversity and Climate Change (https://plone.jcu.edu.au/researchatjcu/research/ctbcc) species database [27]. Species distribution models were fitted using the presence-only approach implemented by the Maxent software [33]. Maxent is particularly suited for presence-only data, often outperforming commonly used statistical methods (e.g. GLM, GAM)[34]. Furthermore, it has shown to give robust predictions for species in this region [27, 35]. Species distribution models incorporated baseline climate data, averaged across 1976 to 2005, at 0.01 degree (~1x1km) resolution. Climate data were accessed from Australian Water Availability Project [36, 37], and the bioclimatic variables were derived using the “climates” package in *R* [38]: annual mean temperature, temperature seasonality, maximum temperature of the warmest period, annual precipitation, precipitation of the driest period, precipitation of the wettest period and precipitation seasonality. We used a target-group as background [39], which consisted of the locations of all the occurrence records for all the species within the class (amphibians, birds, mammals and reptiles) to account for any potential spatial bias in the occurrence data [40].

Distribution models with low performance, assessed with the area under the receiver operating characteristic curve values (AUC<0.7) or poor representations of the species’ known range, were not included in further analyses. Representation of known range was assessed using relevant field guides [41–44], online databases (http://www.arod.com.au/arod/) and expert opinion. Modelled suitable areas within a state or bioregion (Interim Biogeographic Regionalisation for Australia, Version 7) [45] in which the species was known not to occur were clipped out.

Projections of future climate were sourced from the Tyndall Centre (http://climascope.wwfus.org/). Eighteen general circulation models (GCMs), and one emission scenario (RCP8.5) were accessed for the years 2055 and 2085. Species distributions were modelled for each combination of GCM, RCP and year. In order to reduce uncertainty in the projections we sought consensus across 18 GCMs [46, 47], calculating the median modelled suitability across the GCMs for each year for each species and used these in further analyses.

To obtain a reasonable estimate of the areas in which species could be expected to be present or absent, the default continuous prediction of environmental suitability provided by Maxent was clipped below an appropriate threshold; areas with suitability scores below the threshold were deemed to have suitability too low for the species to occur. For each species, four different thresholds obtained from the Maxent results output file were investigated, and the threshold providing the best fit to the species’ known range was selected. Areas below the threshold were given zero suitability; above the threshold the continuous suitability surface was retained. To provide more realistic estimates of species’ future distributions, future projections of suitable climate space were clipped (given a suitability score of zero) beyond a reasonable dispersal distance from species’ current distribution. The dispersal distance used was 3km/year for birds and mammals and 0.5km/year for amphibians and reptiles [48]. While restricting future distribution projections to within a reasonable dispersal distance gives a conservative result, generating future distributions that are similar to the current distributions; we wanted the analysis to be as close to a real-world scenario as possible so that ultimately we could maximise the usefulness as management decision-support.

All modelling and post-processing of models was done as part of the CliMAS project, and can be found online (http://climas.hpc.jcu.edu.au/)[31].

There were 662 vertebrate species (69 amphibians, 331 birds, 105 mammals and 157 reptiles) occurring within the region with satisfactory distribution models for inclusion in the prioritisation analysis. Current models for the 662 species, and the median suitability across the 18 output distributions at both 2055 and at 2085 were included in the prioritisation.

### Spatial prioritizations

Zonation is a method and software for ecologically based land use planning that provides a priority ranking, from which arbitrary best or worst fractions of the landscape can be identified ([49], see [50] for introduction and references). It is often used for cost-efficient reserve network design, where many biodiversity features, costs, connectivity and other considerations need to be balanced [16, 51]. Here, Zonation was used to find areas that efficiently cover present and future distributions of species.

Species were weighted by the fraction of their Australian distribution occurring within the study area, giving the highest weight to the narrow-ranged endemics and lowest weight to the widespread species with marginal occurrences in the study area. The parameter *α* of the negative exponential dispersal kernel for each species was calculated as 2/*d*, where *d* = mean dispersal distance estimates in the same units as used the species distribution modelling [52]. For amphibians and reptiles the dispersal distance was estimated as 0.005 degrees (*α* = 2/0.005 = 400); for birds and mammals dispersal was 0.03 degrees (*α* = 2/0.03 = 66.67)[48]. The Additive Benefit Function (ABF) formulation for aggregating conservation value was used. It accounts for all species, weighted for proportional range size, and effectively minimises expected aggregate extinction rates according to species-specific species-area curves [50]. For comparison, the analyses were repeated with the Core-Area Zonation method of aggregating conservation value. Compared to ABF, this method does not allow trade-offs between species and places relatively more emphasis of guaranteeing protection of high-quality areas for every included species, even when that comes with the cost lower average outcome for all species [16](S1 Table).

Four conservation prioritizations were carried out using Zonation: prioritizing areas using 1) species current modelled distributions only (“Current”); 2) species model projections for 2055 only (“2055”), 3) species model projections for 2085 only (“2085”), and 4) species current modelled distributions, 2055 and 2085 projections, and the connectivity between each of the three time periods for each species (“All Time”)[11]. In this case, connectivity refers to the proximity of the species’ distribution in each time point. For example, locations with high suitability for a species at each time point receive high connectivity scores [15].

Uncertainty of future projected distributions was further accounted for using the distribution discounting function within Zonation [53]. For each species and each future time period, we used the standard deviation of habitat suitability taken across the 18 GCMs as an indication of model uncertainty. To give higher priority to areas where model outputs tend to agree about future habitat quality, we subtracted the standard deviation off the median prediction for each species in each grid cell. This balances the uncertainty of species future distributions while still recognising the importance of future habitat suitability [11].

### Comparison of different solutions

A quantitative comparison between pairs of solutions was undertaken, considering each solution as a whole, and then by taking the top 25% ranking areas in each solution. The number of cells within the top 25% ranks that overlapped in each of the four solutions was calculated.

The ability of each of the four solutions to capture the current and future distributions of each species was first evaluated by calculating the area and proportion of each species distribution that fell within the top 25% of each solution. This was done for the Current, 2055, 2085 and All Time solutions.

### Independent evaluation

An independent evaluation of the solutions was conducted by assessing the four solutions for their capacity to account for species that were not included in prioritisations. There were 71 vertebrates within the study region that were too restricted for useful distribution models to be fitted using the 0.01 degree data. The proportion of occurrence points for each restricted species that fell within the top 25% of each solution was recorded. To test for significant differences in the proportion of severely restricted species protected between the solutions, a generalised linear model with a binomial error family was run. A likelihood ratio test was performed on the model output using the ANOVA function with the option of Chi-squared test, using *R* version 3.0.0 [54].

### Species distribution characteristics

To investigate which characteristics of species distributions influenced how well they were captured in the high-ranking cells (top 25%) of each solution, we used Boosted Regression Tree partial dependence plots. Partial dependence plots provide useful visualisation of the effect of each of the variables, assuming the average effects of all the other variables [55, 56]. These plots are informative providing the predictor variables are not strongly correlated. To this end, we removed highly correlated variables from our predictor set (S1 Table). We followed the protocol and adapted the code outlined in Elith et al. [56], and used the ‘gbm’ package version 2.9 [57], and statistical analysis outlined by Sutcliffe et al. [58]. Tree complexity, i.e. the number of splits in each tree, was set at five to prevent over-fitting but to allow identification of interactions between variables [56]. The learning rate for all models was set at 0.01 to reduce the influence of the primary set of trees on the models.

After removing highly correlated variables, the remaining predictor variables were: size (number of cells with modelled suitable environment) of current and 2085 distributions, size of overlap between current and 2085 distributions, mean elevation across current and 2085 distributions, standard deviation of elevation across the current and 2085 distributions, mean and standard deviation of elevation across the current and 2085 distribution overlap, and the proportion of the current distribution that overlaps with the 2085 distribution.

Total deviance explained by the models was calculated following Sutcliffe et al. [58]:

$$TD=\frac{\bar{x}\;TD-CVD}{\bar{x}\;TD}$$

Where *TD* is the total deviance and *CVD* is the estimated cross-validated deviance. The proportion of the total deviance explained by each predictor variable was assessed using partial dependency plots. All analyses were run in *R* version 3.0.0 [54].

## Results

### Comparison of different solutions

**Fig 2:**
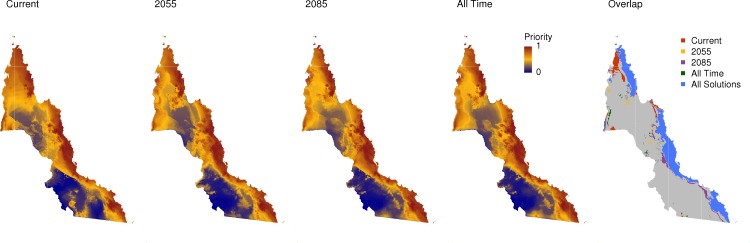
The Zonation solutions for Current, 2055, 2085 and All Time scenarios. The highest ranking cells ranked “1”, shown in red, and the lowest-ranking cells ranked “0”, in blue. Far right: the highest-ranking cells (> top 25%) showing where all solutions overlap, and areas only found in the highest-ranking cells for the Current, 2055, 2085 or All Time scenarios. The grey represents areas that were not in the highest-ranking cells for any solution.

The Zonation solutions for Current, 2055, 2085 and All Time were similar, with the highest ranked cells (i.e. the top 25%) overlapping from 78–85% (Table 1). Each solution prioritized the areas that were upland, close to the east coast and contained rainforest (Fig 2; performance curves shown in Figure A in S1 Appendix). Notable exceptions included areas that were priorities only in the Current solution, which are in the far north of the region. Interestingly, the general patterns differed little when the Core-Area cell removal rule was used (Figure B and Figure C in S1 Appendix), although Core-Area outputs placed higher emphasis on areas away from the east coast, on lowlands and Eucalypt woodlands, particularly in the south of the region.

**Table 1:** The proportion overlap of the highest ranking cells (> top 25%) of each of the solutions.

	Current	2055	2085	All Time
Current	---	0.85	0.78	0.82
2055		---	0.78	0.82
2085			---	0.82
All Time				---

**Fig 3:**
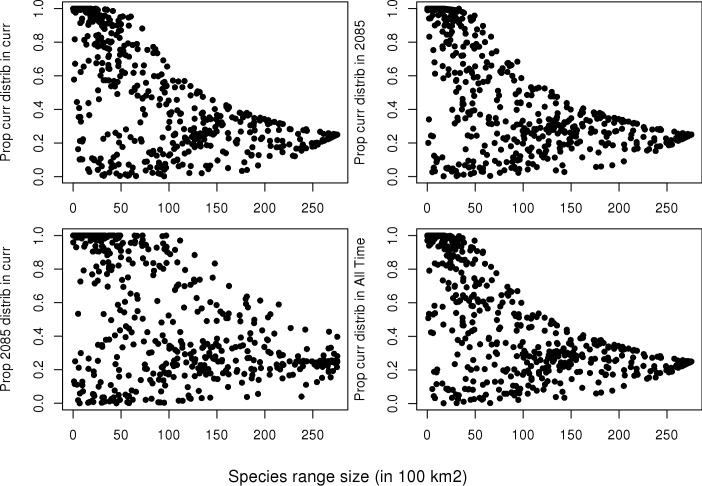
The proportion of species distributions captured by the top 25% of the different solutions plotted against their range size within the region. Top row: 1) proportion of species current distribution within the Current solution, 2) proportion of species current distribution within the 2085 solution. Bottom row: 1) proportion of species 2085 distribution within the Current solution, 2) proportion of species current distribution within the All Time solution.

As expected, species with narrow ranges were more likely to have their entire distribution within the region captured by any solution; this includes species’ current distribution falling within the 2085 solution, and the reverse (e.g. the species’ 2085 distribution falling within the Current solution; Fig 3). Individual species differed in how they were captured by each of the solutions (Figure D in S1 Appendix), with many of the high elevation frog species current and projected future distributions being entirely captured within each of the solutions.

### Independent evaluation

Most of the restricted-range species, for which there was not enough data to model distribution changes under climate change, were accounted for by the top 25% of every solution (Figure E in S1 Appendix). Each solution performed equally well in capturing the restricted species in the high-ranking cells (*df* = 3, likelihood ratio test statistic χ^2^ = 152.71, *p* = 0.99). However, three reptile species were unaccounted for in the top 25% of any solution: Whitsunday rainbow-skink (*Carlia inconnexa*), Australian bockadam (*Cerberus australis*) and Quinkan velvet gecko (*Oedura jowalbinna*). Whitsunday rainbow-skink occurs only on islands that were not included in the prioritisation. Australian bockadam occurs on the western edge of Cape York Peninsula and falls just outside the high priority area in the west. Quinkan velvet gecko occurs on the western edge of the Wet Tropics bioregion, falling just to the west of the high priority region on the east coast. The endangered Golden shouldered parrot (*Psephotus chrysopterygius*) has 4–10% of its current occurrences within the top 25% of each solution; the northern occurrences better accounted for than the south and west of its range. The remaining species with less than half their occurrences accounted for occur outside rainforest, in either savanna, mangroves or coastal woodlands.

### Species distribution characteristics

**Fig 4:**
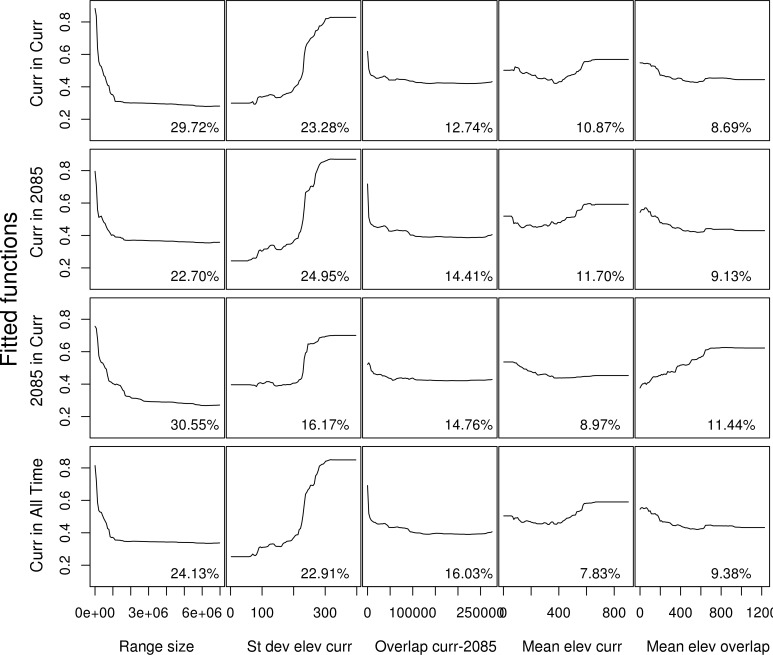
The boosted regression tree partial dependency plots. The plots demonstrate the influence of the predictor variables (columns) on how well species were represented in the highest ranking cells (> top 25%) of each of the solutions. Rows: 1) proportion of species current distribution captured in the Current solution, 2) proportion of species current distribution captured in the 2085 solution, 3) proportion of species 2085 distribution captured in the Current solution, and 4) proportion of species current distribution captured in the All Time solution.

The main factors determining how well current and projected future species’ distributions were captured in each solution were primarily the size of each species’ global distribution, the variation in elevation within the species current distribution (i.e. a proxy for topographic ruggedness), the overlap between species current and projected 2085 distributions (i.e. the nestedness of the future distribution within the bounds of the current distribution), and the mean elevation across species current distribution (Fig 4). The contribution of each of the variables to each model is shown in Table A in S1 Appendix. Overall, small-ranged species with large variance in elevation and species at higher elevations were more likely to be better represented in all solutions. There was little variation of the total mean deviance explained across the models (Table 2).

**Table 2:** The total mean deviance explained, mean residual deviance, estimated coefficient of variation (CV) of deviance, and the number of trees in each of the Boosted Regression Tree models.

	Total Mean Deviance	Mean Residual Deviance	Estimated CV Deviance	Number of trees
Current in All Time	0.293	0.034	0.064	50
Current in Current	0.300	0.043	0.069	50
Current in 2085	0.294	0.035	0.067	50
2085 in Current	0.325	0.041	0.069	50

## Discussion

Fortunately for the Wet Tropics NRM cluster region, there is not a lot of conflict between conservation prioritizations for the current and for the future. This is because of the nestedness of current and future distributions of the narrow-ranged endemic species in the region. Both the current distribution and future distributions of narrow-ranged endemic species at high elevations were well represented within the highest-ranking cells in each of the planning solutions. This is driven by the emphasis Zonation places on small distributions via the range-size normalization and by the large overlaps in these species' current and future distributions in the mountainous areas, and subsequently reflected in the large overlap between planning solutions. This result remained consistent despite different parameters in Zonation, either the Additive Benefit Function or the Core-Area cell removal. Present results support the widespread notion that mountainous areas are important as climate change refugia, both for species that are there currently and for the species projected to find suitable climate space there in the future [40, 59–62]. Furthermore, it reduces the challenge of which time point to plan for. It is encouraging that for the species of most concern in this region–the small ranged endemics–planning for their conservation using either just their current distributions, just their future distributions, or an attempt to incorporate both will all result in similar conservation recommendations.

However, the Current, 2055 and All Time solutions tended to place more emphasis on the north and west of the study region. The Current solution favoured smaller patches throughout the region that were not favoured at other time points. In contrast to the Current solution, few areas were suitable only for the 2085 solution, but the 2085 and All Time solutions overlap in the south.

Interestingly, across all species, there was no difference between the Current solution’s mean ability to capture species’ current distributions and the 2085 solution’s ability to do so. However, the variance among species was substantial, demonstrating that the top ranking cells of each solution varied markedly in how well they captured individual species.

Planning species conservation under future climate change presents inherent uncertainties, and inaccurate predictions might pose a risk to the efficacy of conservation action. The uncertainties propagate at every step of conservation planning under climate change: the physical changes in climate and habitat that might occur, species’ responses to these changes, the consequences of changing species assemblages, and the most effective strategy for conservation across time. The presence of uncertainty should not prevent conservation planning, but rather be accounted for to enhance the robustness of plans to unexpected changes in circumstances [63]. These uncertainties are precisely the reason why comparing current and future conservation prioritisations is warranted. Uncertainty begins with the unknown concentrations of greenhouse gas emissions that will eventuate during the planning horizons [64], the spatial manifestation of changes in climate [65], and propagated through the different methods of downscaling the coarse General Circulation Models (GCMs) to reflect the spatial heterogeneity of climate at the local scale [66]. Species distributions modelling algorithms can produce widely varying outputs [11], and are influenced by the quality and resolution of the input data [67], dispersal scenario employed [20, 68], and the model algorithm’s ability to project into novel environmental space. The output distribution model is often interpreted by assuming that modelled suitability equates with a species’ occupancy, and usually ignores temporal fluctuations in suitability and occupancy [69]. Importantly, even if the physical realities of climate change were known and species models were robust and useful proxies for the broader biodiversity, the actual responses of species to climate change will be determined by factors such as habitat availability and quality, dispersal rate, species interactions, abundance, breeding rate and other population dynamics [70]. Bringing these species distributions models into a conservation planning framework assumes that representation of species (i.e., a grid cells modelled to be suitable currently and in the future) is a useful first proxy for the persistence of species [71]. Then, adding many species and taxa into analysis will ensure coverage of diverse and high-quality environments that should cover and support most species both now and in the future. Many conservation planning studies use only a small number of taxonomic groups, either plants or vertebrates, and implicitly assume that these will be useful surrogates for broader biodiversity value [71]. Naturally, analyses could be repeated periodically following future improvements in data and more reliable information about the progress of climate change in order to adopt a somewhat adaptive strategy to the development of conservation efforts. Finally, conservation planning priorities are influenced by the resolution of the data and the analyses, with relatively fine-scale analysis required for direct linkage with land use decisions [72].

Despite the limitations, research has shown that i) useful insight can be gained by using these uncertain modelling methods, ii) there are established methods for documenting and accounting for the uncertainties, and iii) systematic conservation planning can be a useful tool for strategic conservation, and allow for uncertainties, trade-offs, and transparent decision-making [73,74]. For example, birds have been found to be tracking their niche as projected [2], and future climate change as estimated across a range of GCMs reflects recent realised changes in climate [3, 65], giving confidence that projections can be meaningful. Accounting for uncertainty can be done through judicious choice of greenhouse gas emissions projections, GCMs, downscaling scale and techniques, species distribution algorithms, species input data, dispersal assumptions and interpretation of the outputs. In the current study, we have taken the following measures: used the greenhouse gas emissions projections that the globe is currently tracking; therefore a very likely scenario. Further, it enabled us to examine the conservation plan robustness for the most extreme climate change future. Secondly, we modelled species distribution projections at a fine resolution for a wide range of GCMs (18), and took a median across these. We downweighted species future projections by the uncertainty of the projection in the conservation planning process, so that areas that have high suitability for species and high certainty would get a higher ranking than those areas in which the future suitability is highly uncertain. We used the species distribution modelling algorithm (Maxent) that has shown to have high performance for species models [34], used taxonomic-group specific target-group background points [39] to increase the performance of the models, invested in extensive model vetting, and used taxonomic-group specific dispersal scenarios. These methods have resulted in robust models for species in this region [75]. Here, we used a conservative approach to understand future distributions of species, because many of our species are narrow-ranged endemics restricted by geographic barriers [25, 27, 28, 30].

The similarity of the planning solutions for each time point was expected, because of the geography of the region, the biological patterns, but also because Zonation automatically accounts for species range sizes and places increasing emphasis on occurrences of smaller range species. Furthermore, we weighted species by their global range size, so that species that had distributions widely beyond our study region had lower weights than species that only occurred within this region. This is in contrast to studies in other regions, where large differences were found for current versus future conservation prioritizations in Europe [11, 12] and Japan [13]. Widespread species in the drier lowlands were therefore disadvantaged by these planning solutions in multiple ways: Zonation deprioritised them, we afforded them low weightings, and their suitable climate space was projected to move substantial distances in the future with often only a small proportion overlapping with their current distribution [20, 40]. Constraining species’ future distribution projections within a reasonable dispersal estimate also leads to similar planning solutions for the different time periods. It was necessary to use the constrained projections in this analysis in order to produce conservative, realistic solutions that facilitate decision making by the Natural Resource Managers of this region. Species distribution model projections into the future with unconstrained dispersal scenarios are unlikely to be realistic for many of these species. However, further research could investigate the trade-offs between years using unconstrained future distributions.

Optimising conservation of the widespread lowland species could be achieved by examining these species separately, and possibly in context of their entire distributions and geographic context. Using the current method, their conservation requirements are overshadowed by the small ranged endemics. However, for many of these species less concern is warranted because of the wide variety of environmental tolerances [23], or because of the large amount of woodland habitat still available to them. These species are likely to benefit more from landscape-level habitat management rather than from representation in protected areas within this region.

There is strong recognition that conservation plans and actions need to be adapted to account for climate change [24, 61, 76,77]. Central to this is whether current protected areas will account for the important areas of biodiversity in the future. There are predictions that current protected areas will underperform in the future [7], or become less effective [8, 9]. However, there is evidence that current protected areas will perform better than unprotected areas for conserving biodiversity into the future [4, 5]. The tendency for disproportionate representation of high elevation areas of rugged topography in the protected area networks is advantageous for species tracking their climate space upslope, and for the endemic species that are often found in this terrain [78]. However, for the species currently depending on the mountain tops, protected areas will be unable to ameliorate the complete loss of suitable climate space. Alternatively, ex-situ conservation action might be required for these species [77]. Overall, areas that are currently unprotected but of high conservation value for species both now and in the future should be prioritised for new protected areas and restoration where required. The challenge is to identify the regions in which new strategies are required to ensure species long-term persistence.

## Supporting information

S1 Table The correlation between each of the predictor variables. (XLSX)

S1 Appendix This supporting file contains tables and figures: S1 Appendix Table A.S1 Appendix, Figure A.S1 Appendix, Figure B.S1 Appendix, Figure C.S1 Appendix, Figure D.S1 Appendix, Figure E.
(DOC)
