# Supplementary material for: Examining current or future trade-offs for biodiversity conservation in north-eastern Australia
Source: PLoS One. 2017 Feb 21;12(2):e0172230. doi: 10.1371/journal.pone.0172230 (PMC5319782; doi:10.1371/journal.pone.0172230)
Supplement: S1 Appendix — Table A.S1 Appendix, Figure A.S1 Appendix, Figure B.S1 Appendix, Figure C.S1 Appendix, Figure D.S1 Appendix, Figure E. (DOC) [file pone.0172230.s002.doc]

**Biodiversity trade-offs of planning for current or future**

## Supporting Information

April E. Reside, Jeremy VanDerWal, Atte Moilanen, Erin M. Graham

**S1 Appendix Table A**. The contribution of all variables to the Boosted Regression Tree models

|  | C-AT | C-C | C-2085 | 2085-C | 2085-AT |
| --- | --- | --- | --- | --- | --- |
| Global range size | 24.13 | 29.72 | 22.70 | 30.55 | 29.76 |
| Overlap current-2085 | 16.03 | 12.74 | 14.41 | 14.76 | 14.09 |
| Mean elevation current | 10.70 | 10.87 | 11.70 | 8.97 | 7.83 |
| Mean elevation overlap | 9.38 | 8.69 | 9.13 | 11.44 | 11.87 |
| % overlap | 7.20 | 6.13 | 7.11 | 7.15 | 9.72 |
| Sd elevation current | 22.91 | 23.28 | 24.95 | 16.17 | 15.35 |
| Sd elevation overlap | 9.64 | 8.57 | 10.01 | 10.96 | 11.38 |

**
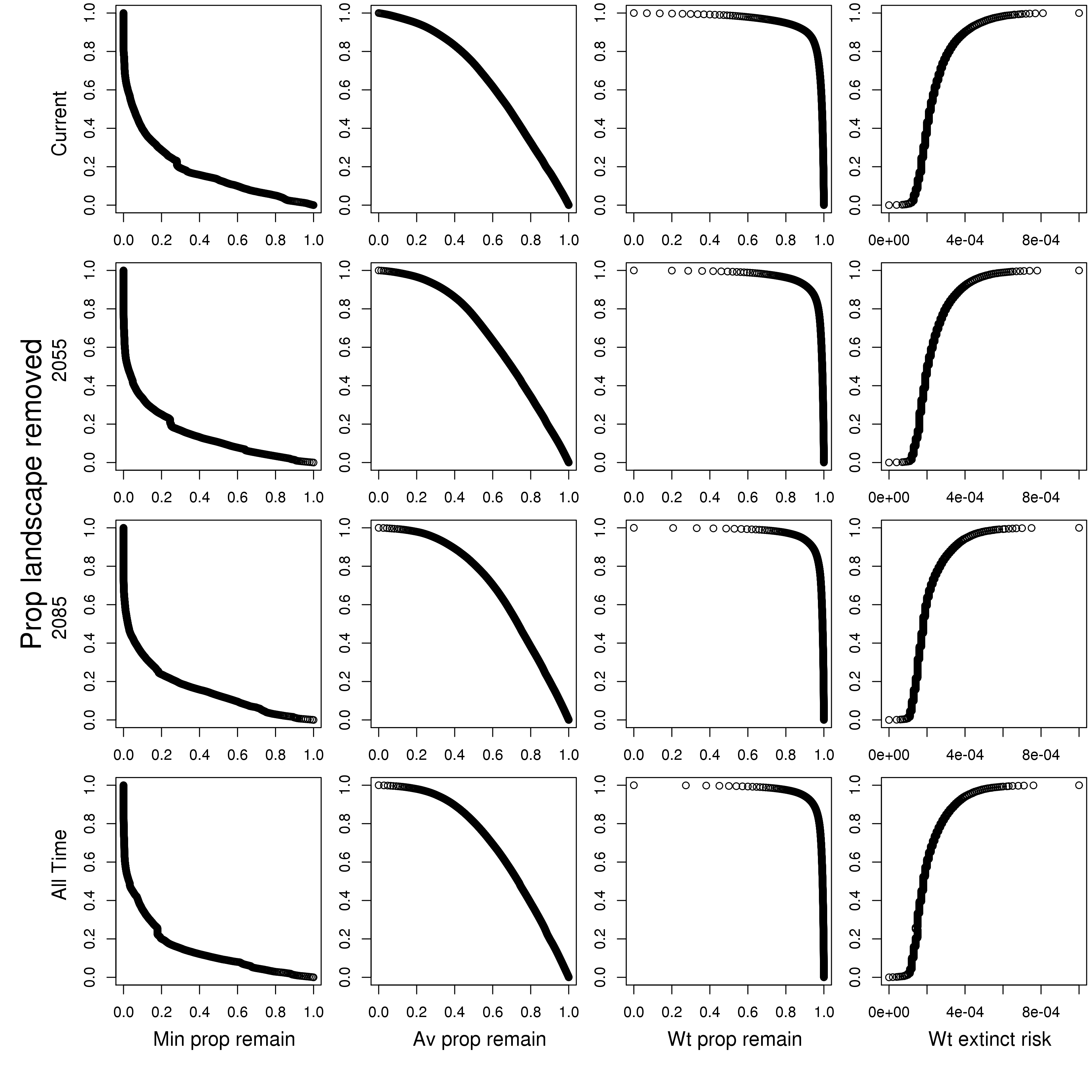
**

**S1 Appendix Fig A. Performance curves from the Zonation analysis showing for each stage of cell removal (y-axis).** a) Minimum proportion remaining of any species, b) the average proportion of species’ distributions remaining, c) the proportion of species distributions remaining, weighted by the species weights and d) the weighted extinction risk.


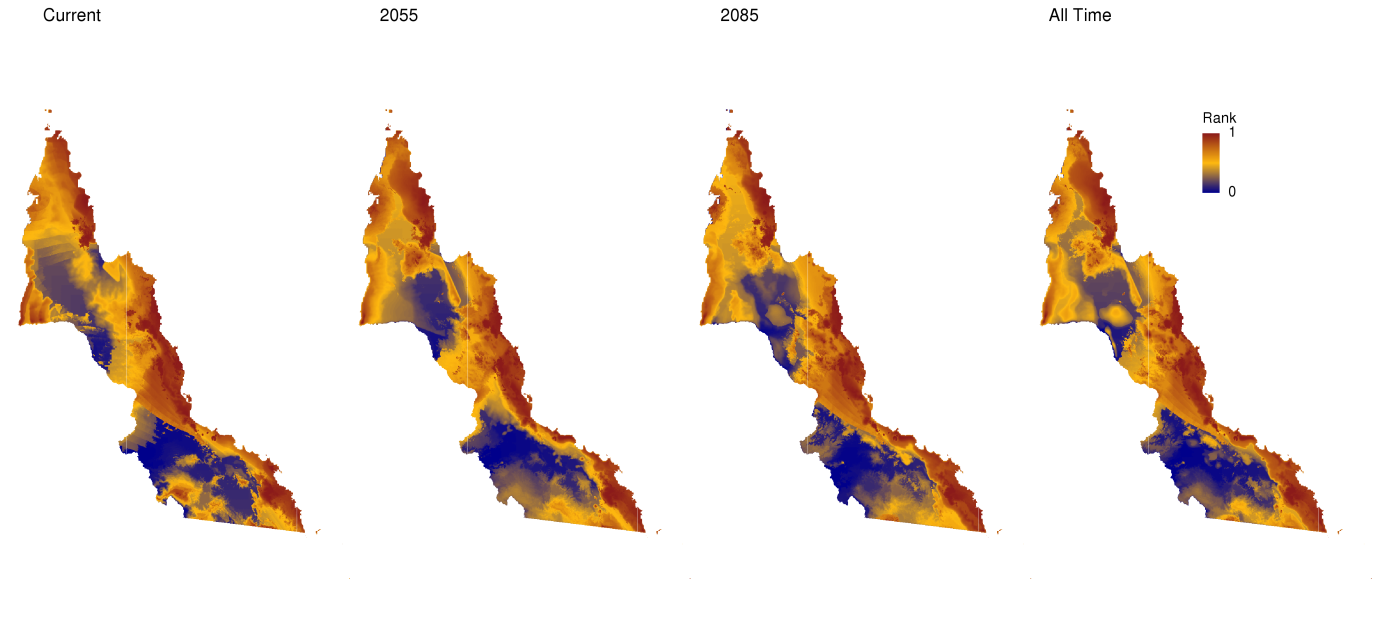


**S1 Appendix Fig B. Zonation solution with Core area cell removal rule for species current distributions; and distributions for 2055, 2085 and All Time.**


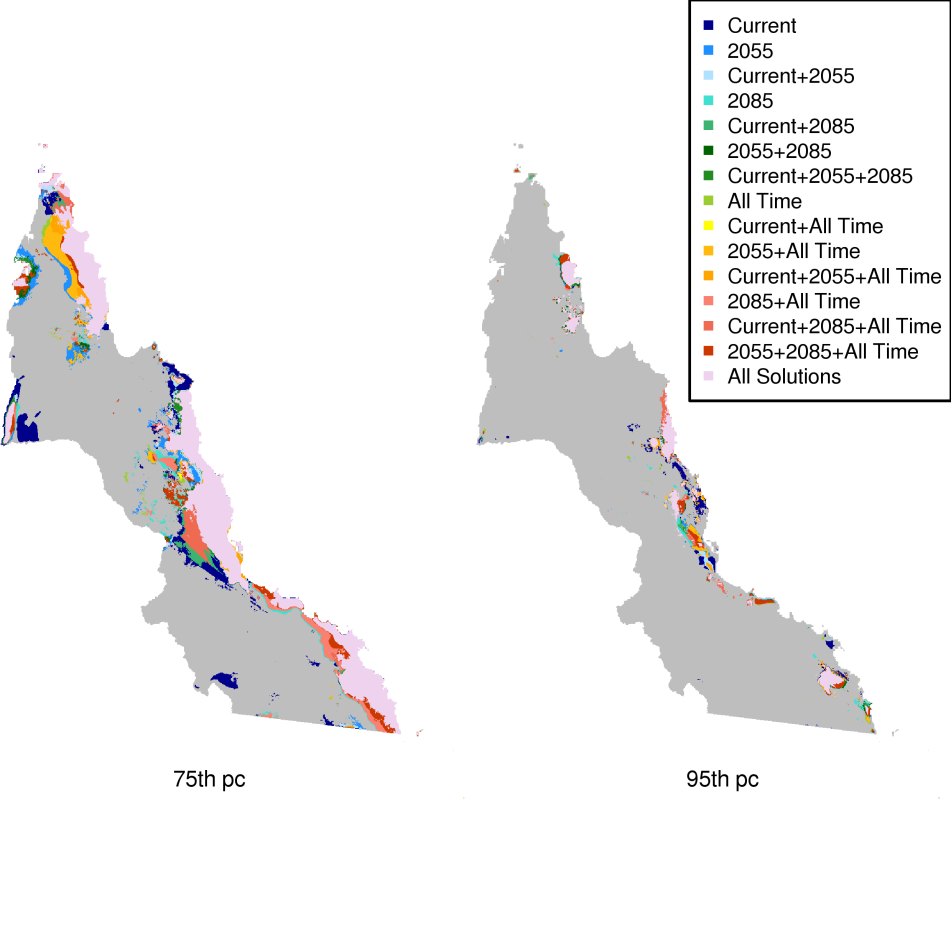


**S1 Appendix Fig C. The differences between the four Zonation solutions (Current, 2055, 2085 and All Time) highlighted for the top ranking areas (75th, left and 95th percentiles, right) using the Core-Area cell removal rule.** Dark blue indicates areas where all four solutions agree; pale blue indicates areas where all but the current solution agree, and pink indicates areas of top ranking for the current solution only.

**

**

**S1 Appendix Fig D. The mean area (left column) and proportion of area (right column) of species current distributions captured in the highest ranking cells (> top 25%) of the four solutions.** Error bars represent standard deviations. From rows top to bottom: amphibians, birds, mammals and reptiles.


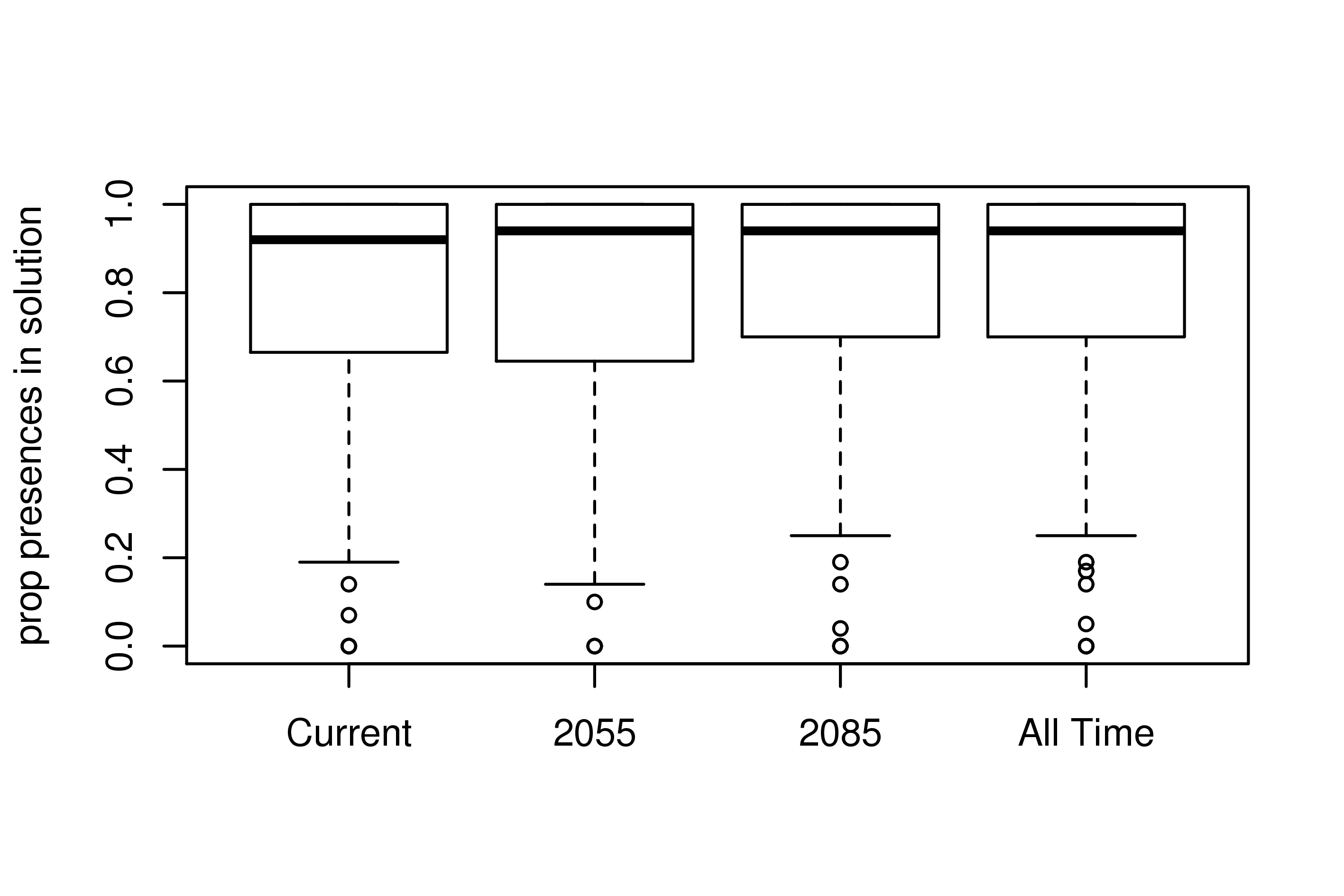


**S1 Appendix Fig E. Proportion of presence data of the restricted species that were accounted for by the highest ranking cells (> top 25%) of each solution in the independent evaluation.**
